# Data Day to Day: building a community of expertise to address data skills gaps in an academic medical center

**DOI:** 10.5195/jmla.2017.35

**Published:** 2017-04

**Authors:** Alisa Surkis, Fred Willie Zametkin LaPolla, Nicole Contaxis, Kevin B. Read

## Abstract

**Background:**

The New York University Health Sciences Library data services team had developed educational material for research data management and data visualization and had been offering classes at the request of departments, research groups, and training programs, but many members of the medical center were unaware of these library data services. There were also indications of data skills gaps in these subject areas and other data-related topics.

**Case Presentation:**

The data services team enlisted instructors from across the medical center with data expertise to teach in a series of classes hosted by the library. We hosted eight classes branded as a series called “Data Day to Day.” Seven instructors from four units in the medical center, including the library, taught the classes. A multipronged outreach approach resulted in high turnout. Evaluations indicated that attendees were very satisfied with the instruction, would use the skills learned, and were interested in future classes.

**Conclusions:**

Data Day to Day met previously unaddressed data skills gaps. Collaborating with outside instructors allowed the library to serve as a hub for a broad range of data instruction and to raise awareness of library services. We plan to offer the series three times in the coming year with an expanding roster of classes.

## BACKGROUND

Recent years have seen significant growth in the data-related services that health sciences libraries offer, with those services often centered on research data management education [1–4]. More recently, these offerings have extended to classes in data visualization [5, 6] and other data science topics [7], but limitations in resources and expertise have restricted the scope of offerings at most institutions. One means of providing a broader range of classes has been to bring in outside instruction, such as the “Data Carpentry” workshops, which provide instruction in the skills and tools needed to work effectively with data [8], although bringing in outside educators can be costly. Other libraries have made use of local expertise to offer workshops through the library [9, 10], either as a monthly series [10] or as an ongoing collaboration with a set of data experts [9].

The New York University Health Sciences Library (NYUHSL) began developing data services in 2011. By 2016, the NYUHSL data services team had expanded to included three faculty who split time between data services and liaison responsibilities and one full-time staff person dedicated to research data management [11]. Classes in research data management have been a core element of these services. These classes have mainly been offered through academic departments, research groups, and training programs (e.g., Clinical and Translational Science Award [CTSA] training programs, graduate curriculum), with outreach conducted by the administrators of those units. Attendance varied widely, ranging from a low of 3 to more than 60 attendees. In 2015, our focus expanded to include services for data visualization, including an introductory “Data Visualization 101” class and another class on tailoring visualizations for different audiences. The Department of Population Health, a particularly library-engaged group, was the primary driver of the development of data visualization training, but we did not know the extent to which this content would interest others at the medical center.

Strategic planning efforts in the library had revealed a widespread lack of awareness of many available library services, including these data classes. The liaison work of the data services faculty, as well as that of others in the library, had provided indications that the demand for data-related educational content outstripped the number and range of offerings that were broadly available at the medical center. While we did not have the range of skills to meet all types of data skills gaps, we were aware of many other people at our institution with data expertise. Many of these people were already teaching data-related classes in existing training programs, but that content was not available to those in the medical center outside of the training programs.

## STUDY PURPOSE

The NYUHSL data services team planned to offer several classes to the New York University Langone Medical Center (NYULMC) community. We approached a number of people with data expertise in the medical center and requested that they teach classes in their areas of expertise as part of a month-long series of data classes at the library. In this way, we could provide a breadth of data-related classes beyond what the library alone could offer, thus establishing the library as a data hub. All were willing to participate, most using preexisting material developed for training programs. Our goals in hosting this series of data classes in the library were to gather information about data skills gaps at the medical center, provide training for those gaps, increase awareness of library data services, and facilitate connections to data expertise across the medical center.

## CASE PRESENTATION

Our interest in finding a way to address data skills gaps at the medical center and to increase visibility of the library’s data-related classes and services coincided with the opening of a new library space. After the NYUHSL was destroyed by Superstorm Sandy in 2012 [12], a new, redesigned library opened in 2016 in a location in the heart of the medical center and included a twenty-four-seat, state-of-the-art classroom. The medical center community’s interest in the new library space, coupled with the availability of the classroom, provided a particularly high-profile opportunity to offer the data class series.

### Content

The first classes scheduled for the series were our preexisting introductory classes on research data management and data visualization, and a second data visualization class on communicating to different audiences. Additional classes on data wrangling, qualitative data analysis, big data in medicine, and clinical research data management were taught by experts from the Institute for Innovations in Medical Education (IIME), a unit focused on educational technology and instructional design for the School of Medicine, the Department of Population Health, and DataCore, a clinical research data services unit at the medical center. An additional library-taught class was added to raise awareness and facilitate the use of i2b2 [13], a tool for identifying clinical research cohorts, and REDCap [14], an electronic data capture tool. In all, the series consisted of eight classes taught by seven instructors from four different units, including three different librarian instructors (Table 1).

**Table 1:**
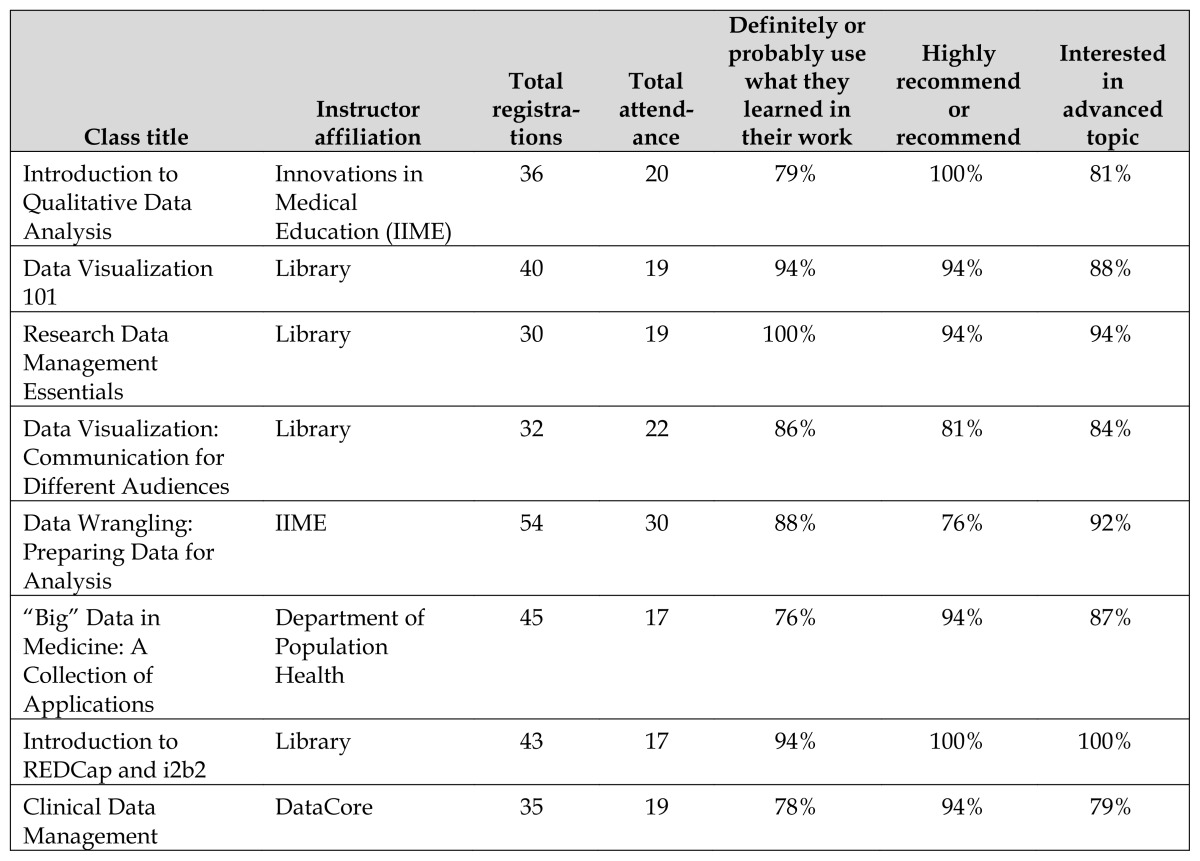
Class title, instructor, registration, attendance, and quantitative evaluations

Class title	Instructor affiliation	Total registrations	Total attendance	Definitely or probably use what they learned in their work	Highly recommend or recommend	Interested in advanced topic
Introduction to Qualitative Data Analysis	Innovations in Medical Education (IIME)	36	20	79%	100%	81%
Data Visualization 101	Library	40	19	94%	94%	88%
Research Data Management Essentials	Library	30	19	100%	94%	94%
Data Visualization: Communication for Different Audiences	Library	32	22	86%	81%	84%
Data Wrangling: Preparing Data for Analysis	IIME	54	30	88%	76%	92%
“Big” Data in Medicine: A Collection of Applications	Department of Population Health	45	17	76%	94%	87%
Introduction to REDCap and i2b2	Library	43	17	94%	100%	100%
Clinical Data Management	DataCore	35	19	78%	94%	79%

### Outreach

When marketing the classes, we promoted the series as a single event rather than a series of disparate lectures. To that end, classes were held at the same time every Tuesday and Thursday for a month, refreshments were provided, and some thought went into coming up with a memorable name. Once the series was dubbed “Data Day to Day,” marketing materials were designed with a visual theme that echoed the nature of the series (supplemental Appendix A).

We used several different avenues for outreach to the medical center community, with upticks in registration noted after each email, newsletter, or presentation. We disseminated information about the series through a presentation at a Department of Population Health faculty meeting; Department of Population Health, Office of Science and Research, Clinical and Translational Science Institute, and NYULMC online newsletters; a poster in the main medical center walkway; and direct outreach to liaison communities, including medical students, basic science graduate students, the Department of Emergency Medicine, the Department of Radiology, and the Division of General Internal Medicine.

### Attendance

All classes were fully registered with a waiting list. The registration cap was initially set at the room capacity of 24 but was increased to 28 to allow for no-shows. The total number of interested individuals—defined as the number of registered, cancelled, and waitlisted individuals—averaged almost 40 per class (Table 1). This total comprised 120 unique individuals who signed up for an average of 2.6 classes each. The average attendance per class was 20.25, with 71 unique individuals, each attending at least 1 class.

Demographic data elements were collected for registrants and attendees from three sources: self-reports of school, departmental affiliation, and role from paper evaluation forms; names and institutional unique identifiers from the online registration system; and names from paper sign-in sheets used in the classroom. There were several limitations to this demographic data. First, evaluation form responses were not always complete. Second, the medical school, but not the hospital, was included as an option, which might have resulted in attendees with a hospital affiliation being undercounted. Third, evaluation forms were anonymous, so unique attendees could not be determined, resulting in demographic data for those attending multiple classes being counted once for each class attended. Finally, matching attendees’ names from the paper sign-in sheet or online registration system to their affiliation was hampered by a lack of complete information in available sources of data about medical center personnel.

Despite these shortcomings, the demographic data revealed several points of interest. First, based on 153 evaluation form responses for attendees’ affiliations (by which attendees migh be counted multiple times), the following departments had the highest attendance numbers: Population Health (18), IIME (12), Radiology (11), and Emergency Medicine (10). There were 28 instances of students and 33 instances of summer interns attending the classes. While we had conceived of the class as a resource for researchers, several other types of individuals also attended, including 6 administrators, 2 members of Medical Center Information Technology (MCIT), 6 nurses, and 6 members of the patient experience department. Sign-in sheets showed that the most popular classes for those with hospital affiliations were on data visualization and qualitative data analysis.

### Assessment

Attendees evaluated each class using the paper evaluation form distributed at the beginning of class and collected at the door at the conclusion of class (supplemental Appendix B). These forms assessed the following using Likert-type scales: effectiveness of instructor, level of material, length of the class, whether the attendee would recommend the class, and whether the attendee would use the material from the class. Additionally, 4 free-text fields allowed attendees to provide feedback on the following: course expectations, interest in advanced topics, interest in new topics, and general comments. We received an average of 19.25 evaluations for each class (95% response rate), although not all evaluations were complete.

All classes received positive ratings (Table 1). Across all classes, 87% of respondents indicated that they probably or definitely would use what they learned in that class in their work, 91% indicated that they would either highly recommend or recommend the class, and 92% indicated that they found the instructors to be either highly or mostly effective. Most respondents felt that the level of material presented was “just right” (80%) and that the time allotted was “just right” (85%). The majority of respondents (88%) were interested in more advanced topics in the area of the class that they took. Supplemental Appendix C provides full evaluation data.

A single reviewer coded attendees’ free-text responses to questions on their interest in advanced or new topics. After noting that many participants used these two fields interchangeably, we decided to group these responses together for coding purposes, which resulted in some duplication of responses. In addition, because we pooled responses across classes, duplication of responses may have resulted from single individuals giving a similar response in multiple classes. Codes were iteratively created to reflect themes in attendee comments, with 3 broad themes emerging: interest in in-depth exploration of topics or instruction in a new topic (56 responses), interest in training in specific software or tools (46 responses), and interest in hands-on workshops related to the current class (8 responses). The most requested topics for further instruction were research methods (25 responses) and visualization topics (12 responses), and the most requested training on software or tools was R programming (15 requests) and REDCap (13 requests) (Table 2). Appendix D provides detailed coded responses to these questions.

**Table 2:**
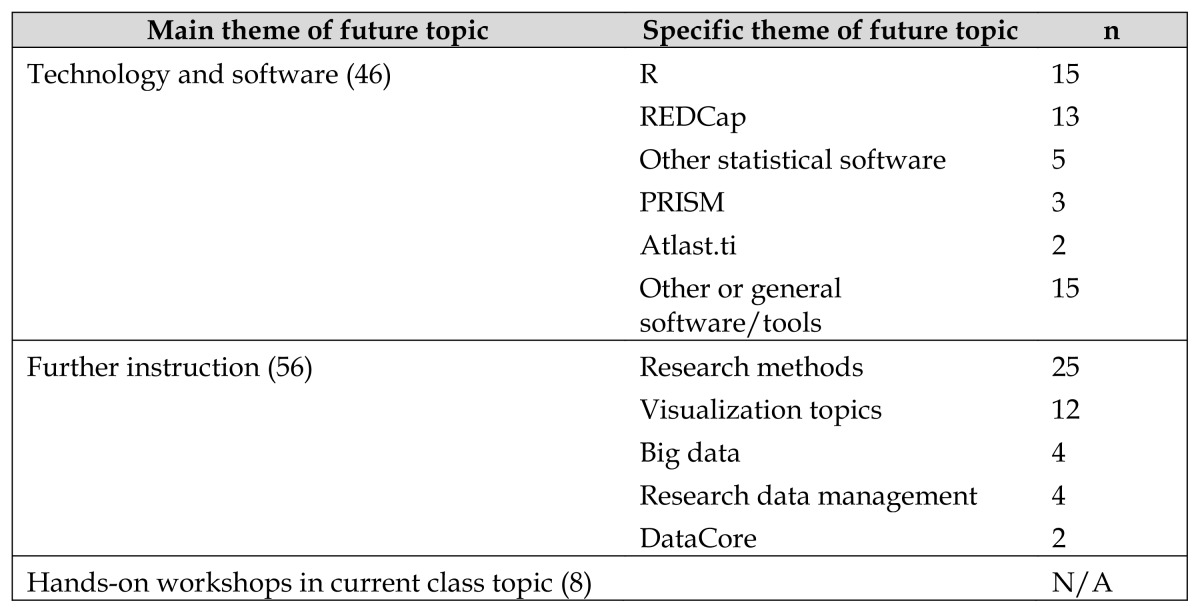
Interest in future topics by theme

Main theme of future topic	Specific theme of future topic	n
Technology and software (46)	R	15
	REDCap	13
	Other statistical software	5
	PRISM	3
	Atlast.ti	2
	Other or general software/tools	15

Further instruction (56)	Research methods	25
	Visualization topics	12
	Big data	4
	Research data management	4
	DataCore	2

Hands-on workshops in current class topic (8)		N/A

Coded responses to free-text evaluation questions on interest in future classes with frequencies in parentheses.

Overall, attendees had positive comments for each class. Seven comments included statements thanking the library for the series, and three individuals specifically requested future Data Day to Day events (e.g., “Great series—could not attend all sessions. Would like a repeat.”). Additionally, two comments indicated that the class series helped to promote the medical center and library’s involvement in data services (e.g., “Great service, I did not know was available. Thank you!”). There were no comments critical of the series as a whole. Ten comments pointed out shortcomings in specific classes. Three addressed a mismatch between the learner’s expectations of the class and the type of material presented; two noted an overlap of material between the two data visualization classes; and five concerned various aspects of the data wrangling class, such as the level of the material and the presentation style.

## DISCUSSION

With waitlists for every class offered, our Data Day to Day series clearly met an unaddressed need for data skills training. Using registrations as a means of gauging the data skills gaps at our institution, we found that significant skills gaps existed in all topic areas covered by the classes. These data skills gaps existed alongside a wide range of data expertise, but that expertise was often siloed in training programs. Instructors had developed a great deal of teaching material for those training programs, but it was unavailable to the general community. Creating a means of offering that material across the medical center resulted in meeting data skills gaps with minimal additional effort on the part of instructors.

While we had feared that scheduling the series in the summer would result in low enrollment due to people vacationing and students being away, the timing proved to be advantageous in some respects. The series provided a professional development opportunity for summer interns, our largest user group, during a normally slow time. While not all medical students are onsite over the summer, this was also one of our largest user groups, indicating that those who were onsite had both the time for the classes and a strong interest in the offered topics. Finally, the breadth of attendees indicated that the need for data skills at the medical center was not limited to researchers, as individuals from the hospital, administration, and MCIT participated.

Three of the four departments with the highest attendance were Population Health, Radiology, and Emergency Medicine, all of which have active liaisons and received direct outreach for Data Day to Day, indicating that direct outreach to a user community is highly effective. The fourth department was IIME, a unit with which two of the instructors were affiliated. The uptick in registrations with each new means of outreach indicated that there was no sole means of reaching the entire medical center community. Despite all the avenues of outreach used, we encountered several medical residents who reported not having heard of the series, indicating there were still communities in the medical center that our outreach efforts had not effectively reached.

Attendees’ comments indicated an interest in future series as well as increased awareness of the library as a provider of data services. In the few weeks following Data Day to Day, we received eight requests for consultations and/or classes in research data management, data visualization, i2b2, and REDCap, a number comparable to what we might normally see in a six-month period. This marked increase in consultation requests indicates that our goal of raising the profile of the data services that the library offered was met. Based on the excellent response, we have begun planning the second Data Day to Day series and will offer the series three times in the coming academic year. Plans to expand the network of instructors are already underway, with commitments from three new instructors for instruction in geographic information systems (GIS), advanced qualitative data analysis topics, and biostatistics.

One advantage of this type of programming is that it requires few resources. The costs associated with the series were limited to printing two posters and purchasing light refreshments, neither of which were strictly necessary. The time investment on the part of the library was also small. Instructors from outside the library taught half of the classes, and most classes were taught using previously developed material. Some time was required to develop marketing materials and evaluation forms, but these are now available to be used in future Data Day to Day series. Because of this low overhead, both financially and in time commitment, this format allows low-risk experimentation around content and structure. Even “failures” (i.e., classes with low attendance) provide valuable information as to which topics are of limited interest and so do not warrant inclusion in future series. The information we gathered also helped us gain a better understanding of our user community and thus informed how we should adapt and grow our existing data services.

## Supplemental Files

Appendix A Marketing poster

Appendix B Class evaluation form

Appendix C Full evaluation data

Appendix D Qualitative data coding
