# Supplementary material for: Data Day to Day: building a community of expertise to address data skills gaps in an academic medical center
Source: J Med Libr Assoc. 2017 Apr;105(2):185–91. doi: 10.5195/jmla.2017.35 (PMC5370612; doi:10.5195/jmla.2017.35)

JMLA Journal of the Medical Library Association

Supplemental content to
J Med Libr Assoc. Apr;105(2): dx.doi.org/10.5195/jmla.2017.35
www.mlanet.org
© Surkis, LaPolla, Contaxis, Read 2017

Data Day to Day: building a community of expertise to address data skills gaps in an academic medical center

Alisa Surkis PhD, MLS; Fred Willie Zametkin LaPolla, MLS; Nicole Contaxis MLIS; Kevin B. Read, MLIS, MAS

APPENDIX A

Marketing poster

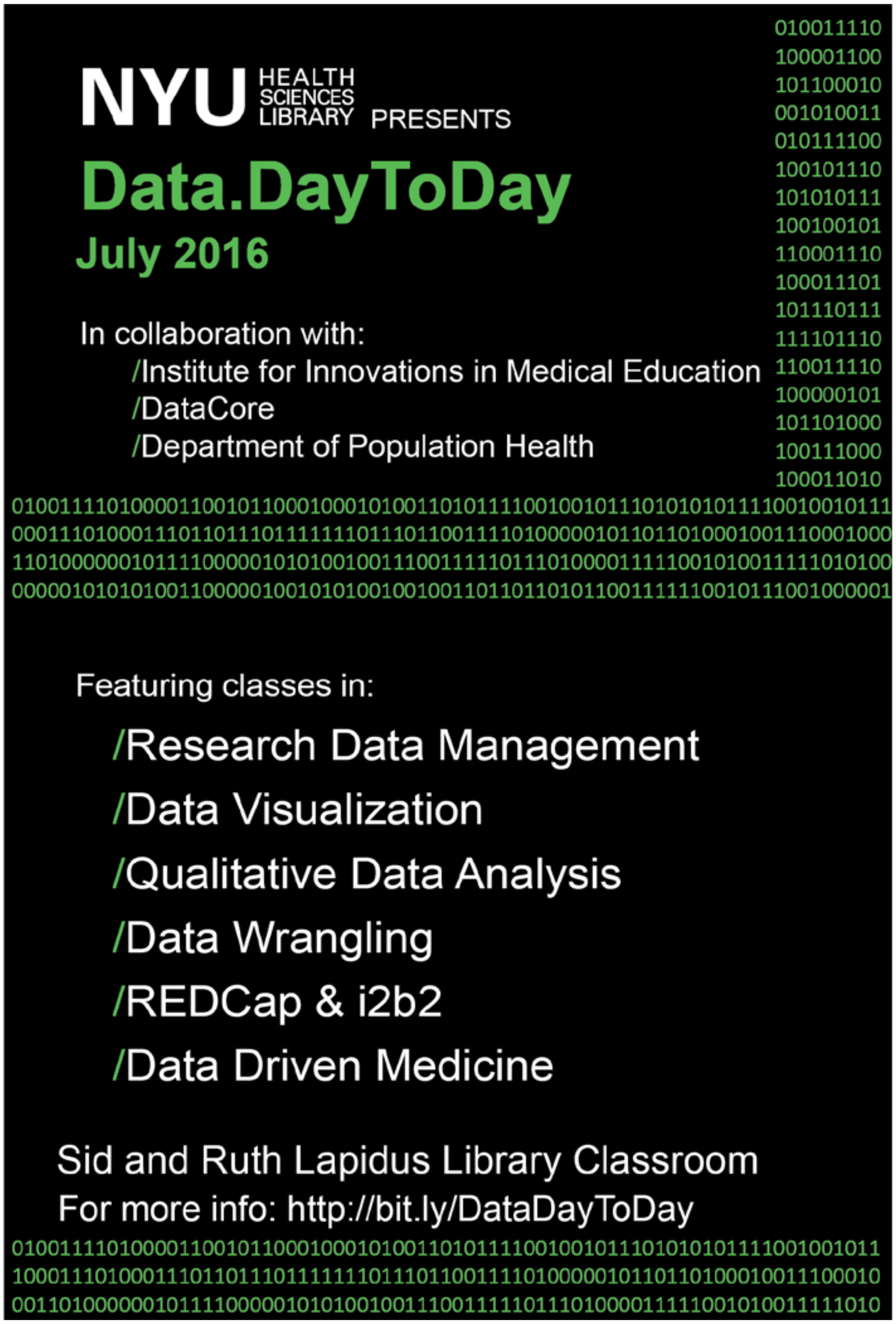

Supplement: Appendix A [file jmla_apr17_surkis_appa.pdf]
