# Supplementary material for: Data Day to Day: building a community of expertise to address data skills gaps in an academic medical center
Source: J Med Libr Assoc. 2017 Apr;105(2):185–91. doi: 10.5195/jmla.2017.35 (PMC5370612; doi:10.5195/jmla.2017.35)
Supplement: Appendix B [file jmla_apr17_surkis_appb.pdf]

JMLA Journal of the Medical Library Association

SC

Supplemental content to
J Med Libr Assoc. Apr;105(2): dx.doi.org/10.5195/jmla.2017.35
www.mlanet.org
© Surkis, LaPolla, Contaxis, Read 2017

Data Day to Day: building a community of expertise to address data skills gaps in an academic medical center

Alisa Surkis PhD, MLS; Fred Willie Zametkin LaPolla, MLS; Nicole Contaxis MLIS; Kevin B. Read, MLIS, MAS

APPENDIX B

Class evaluation form

School:
- o School of Medicine
- o College of Dentistry
- o College of Nursing
- o Other: ____________________

Department/Division: ____________________

What is your role (e.g. postdoc, faculty, student, intern, administrator)?

____

What did you hope to get out of this class and to what degree did you get that?

____

Would you recommend this class to others?
- o Highly recommend
- o Recommend
- o Recommend with reservations
- o Not recommend

Will you use what you learned in this class for your work?
- o Definitely will
- o Probably will
- o Probably won't
- o Definitely won't

Was the level of the material presented:
- o Too low
- o Just right
- o Too advanced

Was the length of time allotted for this topic:
- o Too short
- o Just right
- o Too long

JMLA Journal of the Medical Library Association

SC
Supplemental content to
J Med Libr Assoc. Apr;105(2): dx.doi.org/10.5195/jmla.2017.35
www.mlanet.org
© Surkis, LaPolla, Contaxis, Read 2017

Was the material effectively presented?
- o Very effectively presented
- o Mostly effectively presented
- o Somewhat effectively presented
- o Not effectively presented

Would you be interested in more advanced topics in the area of this class?
- o Yes
- o No

If yes, are there any topics in particular you would like to see?

What other topics would you be interested in seeing offered in future Data Day to Days?

Please share any additional comments about the Data Day to Day series or this class:
